# Evidence of mutations in tumour suppressor genes among oral cancer in Naswar, smokeless tobacco users

**DOI:** 10.2340/aos.v84.43778

**Published:** 2025-06-03

**Authors:** Fatima Iqbal, Sajjad Ahmad, Hoor Maryam, Humaira Amin

**Affiliations:** a Faculty of Health & Medical Sciences, Riphah International University, Islamabad, Pakistan; b Department of Genomics and Bioinformatics, Cholistan University of Veterinary and Animal Sciences, Bahawalpur

**Keywords:** Oral squamous cell carcinoma, tumour suppressor genes, whole exome sequencing, mutations, targeted therapy

## Abstract

**Objective:**

Smokeless tobacco has been linked to the genetic modification of oral squamous cell carcinoma (OSCC). Our study aims to further investigate the disease among Naswar users at the genomic level to understand genetic diversity and discover new targeted therapy.

**Methods:**

A multi-centre descriptive cross sectional research was designed comprising a total of 80 cases of OSCC who were habitual users of Naswar. Out of the 80 cases, whole exome sequencing (WES) was applied to 7 formalin fixed paraffin embedded (FFPE) tissues of OSCC. We further investigated immunohistochemical expression of mutant TP53 and CDKN2A protein in tissues of 80 OSCC samples. Statistical analysis was performed using the Statistical Package for Social Sciences (SPSS) version 19.

**Results:**

Among the total 2,216 somatic variants identified in tumour suppressor genes (TSGs), we compared the high frequency mutation genes reported in OSCC in Catalogue of Somatic Mutations in Cancer (COSMIC) database with research samples, and found that TP53 (85.7%), NOTCH1 (85.7%), and FAT1 (85.7%) showed higher rate of mutation. Among single nucleotide variants, higher prevalence of C/T and G/A base change was noted. Interestingly, a distinct panel of 12 genes was detected to be mutated in 100% samples which was not previously reported compared to Single Nucleotide Polymorphism Database (dbSNP). PTPRT mutation (rs2867655) was present in seven samples and IGF2R (rs629849) was seen in two samples. A statistically significant relation was observed between mutant TP53 protein expression and duration of Naswar use and clinical stages while difference in CDKN2A protein expression was found to be statistically significant with respect to stage only.

**Conclusions:**

Our study presented preliminary data of genetic aberrations in patients exposed to known risk factor (Naswar). These findings can enhance the understanding of genetic aetiology and serve as basis for innovative targets of therapy.

## Introduction

Lip and oral cavity cancer is more prevalent in the Asian subcontinent occurring as the 10th most common cancer, with 2.6% incidence and 2.6% death rates. In Pakistan, it is counted among the top three leading cancers and is a primary health concern [1]. Despite the advancements in oral squamous cell carcinoma (OSCC) therapeutic research, there is no improvement in the overall survival rate at 5 years which is still around 50 to 60% [2]. The foremost difficulty hindering the recognition of specific targets is the complex and heterogeneous nature of this disease. In addition, the revelation of new and altered targets is not completely in practice. Unfortunately, the traditional strategies of treatment for the disease are highly toxic, and the outcome of treatment is different, leading to unnecessary side effects ultimately compromising the quality of life of the patient [3].

The genetic background and risk factors are however, reshaping OSCC. In South Asia, smokeless tobacco (SLT) users are about more than 250 million [4]. One survey revealed there are 352 million SLT consumers globally, 90% of these live in 11 countries among which Pakistan constitutes 10.1 million consumers [5]. Naswar is among the most common forms of SLT consumed in Pakistan, and is predominantly used in Khyber Pakhtunkhwa (KPK) province, which is linked to the Pashtun tribes. It is made up of lime, ash, dried tobacco leaf, flavouring agents, and is composed of nicotine, nitrosamines, and other non-combustible carcinogens which are absorbed through mucosa of the oral cavity [6–8]. A strong association between Naswar users and OSCC has been reported in KPK revealing a risk as high as 20 times to develop OSCC when compared to non-Naswar users [9].

The extent of DNA damage in people is associated with their harmful oral habits. People using alcohol and tobacco experience greater DNA damage than those using either of them [6]. Genetic abnormalities occurring in malignant squamous cells vary with type of tobacco use. Non-identical genetic variations were found in Pakistan and India where SLT is widespread while a study conducted in the United States of America (USA) showed different analysis of genes associated with cigarette smoking [10–12]. Oral squamous cell carcinoma caused by other forms of SLT ‘shammah’ and ‘khat’, common among Arabian population, betel quid, and areca nut revealed different candidate genes and cellular pathways not previously reported [13–16].

The variation in mutational landscape of oral cancer is documented; however, risk based assessment of mutations in OSCC is scarce. This study is designed to uncover genetic aberrations underlying OSCC in high risk population. We also investigated mutant TP53 and CDKN2A potential protein expression and their relation with associated clinico-pathological spectrum of OSCC.

## Materials and methods

### Study participants

The study was conducted from 2020 to 2022 on 80 patients of OSCC who had history of consuming Naswar. Out of the 80 cases, whole exome sequencing (WES) was applied to 7 FFPE tissues of OSCC. The biopsies of the patients were referred from oral and maxillofacial surgery departments of four different hospitals to Histopathology Division, Peshawar Medical College Laboratories. The tissue specimen after excision was placed in 10% buffered formalin for 16 h. Haematoxylin and Eosin (H & E) stained sections were prepared to confirm the tissue diagnosis as OSCC by pathology report employing the World Health Organization (WHO) criterion [17]. All those patients who had previously received any treatment or recurrence or OSCC combined with any other malignancies were excluded. Our study was approved by the Institutional Review Board (IRB) of Prime Foundation Pakistan (IRB Approval Number. Prime/IRB/2018-131). Written informed consent was signed by patients who were willing to participate in this study.

### Whole exome sequencing

Whole exome sequencing was performed at Beijing Genome Institute (BGI) under the project ID F23A430002027_HOMotpvX. Qubit2.0 was used to quantify the DNA, and DNA concentration of at least 2 µg was used to build the library. The genomic DNA was randomly fragmented by using Covaris fragmentation apparatus, and DNA of proper size (~300bp) was collected by electrophoresis. For library preparation and to capture exonic regions, v5 kit was used. High throughput sequencing was performed on DNBSEQ platform.

### Parameters for data filtering

SOAPnuke software was used to remove contamination and obtain valid data [18]. The steps of filtering included: (1) Filter adapter: if the sequencing read matches 25.0% or more of the adapter sequence (maximum two base mismatches are allowed), remove the entire read; (2) Filter read length: if the length of sequencing read is less than 150bp, discard the entire read; (3) Remove N if the N content in sequencing read accounts for 0.1% or more of the entire read, discard the entire read; (4) Remove polyX: if the length of polyX (X can be A, T, G or C) in the sequencing read exceeds 50bp, discard the entire read; (6) Filter low-quality data: if the bases with the quality value of less than 10 in the sequencing read account for 50.0% or more of the entire read, discard the entire read; (7) Obtain clean reads: the output read quality value system is set to Phred +33.

### Bioinformatics analysis

Mapping to the indexed reference genome was performed using BWA tool [19]. For marking the duplicates, the aligned BAM files were given as input to Mark Duplicates (Picard) tool [20]. We have used MuTect2 software for the variant calling. It represents highest validation rate (90%) for mutation detection. Data were provided to MuTect2 in tumour-only mode after alignment of the reads to a reference genome and standard preprocessing steps. Panel of Normals (PoN) was also used from the GATK website. Annotation of the variants was performed through ANNOVAR tool [21]. The tumour suppressor genes (TSGs) were filtered according to TSG database (TSGene) [22], and variants were filtered according to Sift, mutational taster pred, and CADD phred scores. The COSMIC (Catalogue of Somatic Mutations in Cancer) database was used to compare high frequency mutation genes in OSCC [23]. The variants not reported in Single Nucleotide Polymorphism Database (dbSNP) were considered as novel (https://www.ncbi.nlm.nih.gov/snp/).

### Immunohistochemistry

Tissue sections of 4 µm thin were prepared from 80 FFPE blocks. Deparaffinisation of the sections was carried out by incubating in two washes of xylene for 10 min each. Then sections were incubated in two washes of 100% ethanol for 5 min followed by single wash of 90 and 70% ethanol for 5 min each. Sections were washed three times in distilled H_2_O for 5 min each until they were clean. Slides were dipped in antigen retrieval solution 10mm sodium citrate buffer pH 6.0 preheated at 95°C for 15 min in water bath, and then were allowed to cool for 30 min at room temperature. The activity of endogenous peroxidase was blocked by adding 3% hydrogen peroxide in methanol for 15 min. Sections were rinsed with ddH_2_0 twice and PBS once for 5 min. Prior to antibody application, sections were blocked in 2.5% normal horse serum (VECTASTAIN ELITE ABC UNIVERSAL KIT) for 60 min followed by rinsing for 5 min in wash buffer (PBS + 0.025% Triton X). Immunohistochemical staining was performed by incubating sections in 200 µl primary antibodies (ab32049) anti-mutant p53 antibody [Y5] and (ab108349) anti-CDKN2A/p16INK4a in the humidified chamber at 4°C overnight, 1 drop of biotinylated 2° AB was added for 30 min, ABC reagents for 30 min, and DAB staining for 5 min at room temperature. Finally the tissues were stained with haematoxylin (diluted 1:5 times) for 1 min and mounted with DEPEX. The slides were observed under microscope (Olympus light microscope) for protein staining using 4x to 40x magnification range. The criteria for positive immunohistochemical expression of mutant TP53 was the presence of brown particles in the nucleus of cells. It was further grouped into the following categories based on the number of positive cells: negative (-) = no brown particles, weakly positive (+) ≤ 30% cells were mutant TP53 positive, moderately positive (++) 30–70% cells were mutant TP53 positive, and strong positive (+++) > 70% cells were mutant TP53 positive [24]. The immunohistochemical evaluation of nuclear and cytoplasmic CDKN2A expression was performed under light microscope at magnification 40x. The specimen was considered CDKN2A positive when high and diffuse nuclear and cytoplasmic staining was observed in > 70% of tumour cells [25].

### Statistical analysis

Statistical analysis was performed using the Statistical Package for Social Sciences (SPSS) version 19. Mean and standard deviations were measured for continuous variable for example, age. Pearson chi-square test was used to assess correlation between categorical variables. Fisher’s exact test was applied where values are less than 5. Probability value of less than or equal to 0.05 (*p* ≤ 0.05) was considered statistically significant.

## Results

### Patients’ data

Whole exome sequencing of total seven OSCC patients consuming Naswar was performed. The clinico-pathological information of the patients is summarised in Table 1. The clinico-pathological information mainly include: age, site, duration of Naswar use in years, degree of differentiation, stage, among others.

**Table 1:** Clinico-pathological information of the patients of whole exome sequencing analysis.

Clinico-pathological variables	Total no of patients (*N* = 7) (%)
**Age in years**	
Mean	58.71
Standard deviation (SD)	± 13.1
Range	30–70
**Gender**	
Male	6 (86.0)
Female	1 (14.0)
**Site**	
Buccal mucosa	2 (29.0)
Alveolar ridge	1 (14.0)
Retro molar trigone	4 (57.0)
**Risk habits**	
Snuff only	7 (100.0)
Snuff with other products	0 (0.0)
**Duration of use in years**	
1–10	1 (14.0)
11–20	2 (29.0)
> 20	4 (57.0)
**Status**	
Present user	6 (86.0)
Ex user	1 (14.0)
**Duration of lesion**	
< 1 month	0 (0.0)
1–6 months	6 (86.0)
> 6 months	1 (14.0)
**Degree of differentiation (WHO)**	
Well	6 (86.0)
Moderate	1 (14.0)
**Stage (AJCC 8th edition)**	
I	1 (14.0)
II	1 (14.0)
III	1 (14.0)
IV	4 (57.0)

### Mutational spectrum

**Figure 1:**
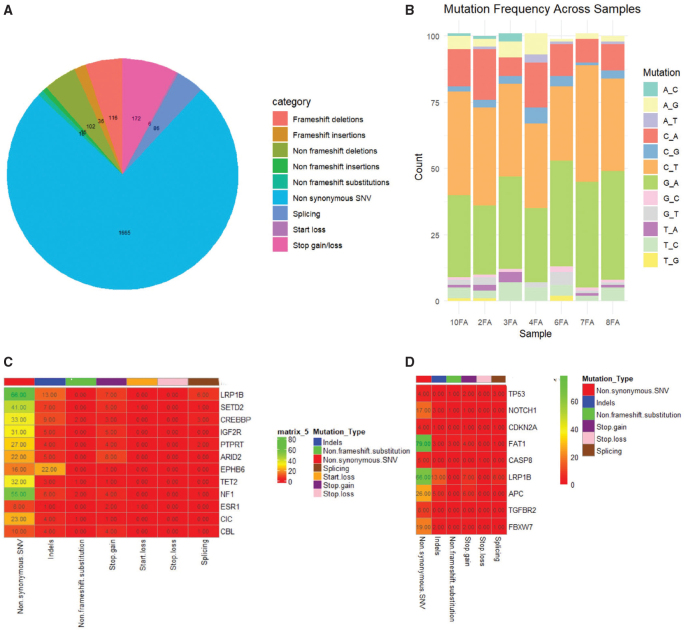
Mutational spectrum of 7 OSCC Naswar users. (A) The proportion of different mutations identified in tumour suppressor genes (TSGs) in 7 OSCC patients. (B) Frequency of single nucleotide change in 7 samples. (C) Somatic variants identified in 12 genes mutated in 7 samples. (D) Somatic variants identified in high frequency mutated genes in 7 samples.

We identified a total of 11,811 somatic variants in all genes of OSCC patients consuming Naswar. The number of mutations in TSGs was counted showing 2,216 of different types of exon mutations and splicing. Non-synonymous single nucleotide variant (SNV) accounted for 1,665. Among indels, we detected 116 frameshift deletions and 36 frameshift insertions while non-frameshift deletions and insertions accounted for 102 and 15, respectively of all exon mutations. Stop gain/loss accounted for 172 of all mutations in TSGs (Figure 1A). In the detection of single nucleotide variants, we found C/T and G/A as the most common types of transitions (Figure 1B). Interestingly, we identified 12 genes including LRP1B, SETD2, CREBBP, IGF2R, PTPRT, ARID2, EPHB6, TET2, NF1, ESR1, CBL, and CIC which harbour mutations in all samples. The somatic variants shown by these genes were non-synonymous SNV, indels, non-frameshift substitution, stop gain, start loss, and splice site (Figure 1C). We also verified the accuracy of our results by comparing the high frequency mutation genes reported in OSCC in COSMIC database with research samples, and found that all TSGs were mutated in patients with variable frequency. TP53 (85.7%), NOTCH1 (85.7%), and FAT1 (85.7%) showed higher rate of mutation which are also ranked in top four in the COSMIC database (Table 2). The type of the mutation exhibited by these genes is shown in Figure 1D.

**Table 2:** Comparison of high frequency mutation tumour suppressor genes of OSCC in COSMIC database and research samples.

Genes	Sample 1	Sample 2	Sample 3	Sample 4	Sample 5	Sample 6	Sample 7	*N* (%)
TP53	Mutated	Mutated	Not mutated	Mutated	Mutated	Mutated	Mutated	6 (85.7)
NOTCH1	Mutated	Mutated	Not mutated	Mutated	Mutated	Mutated	Mutated	6 (85.7)
CDKN2A	Mutated	Not mutated	Not mutated	Mutated	Mutated	Not mutated	Mutated	4 (57.1)
FAT1	Mutated	Mutated	Not mutated	Mutated	Mutated	Mutated	Mutated	6 (85.7)
CASP8	Mutated	Mutated	Not mutated	Not mutated	Not mutated	Not mutated	Mutated	3 (42.8)
LRP1B	Mutated	Mutated	Mutated	Mutated	Mutated	Mutated	Mutated	7 (100.0)
APC	Mutated	Not mutated	Mutated	Not mutated	Mutated	Mutated	Mutated	5 (71.4)
TGFBR2	Not mutated	Not mutated	Not mutated	Not mutated	Mutated	Not mutated	Mutated	2 (28.5)
FBXW7	Mutated	Mutated	Not mutated	Mutated	Mutated	Mutated	Mutated	6 (85.7)

A comparison of mutated TSGs identified in all research samples with the dbSNP was performed. Our study revealed that all of the 12 genes had mutations which were not previously reported. Interestingly, PTPRT mutation (rs2867655) was present in 7 samples and IGF2R (rs629849) was seen in 2 samples. We found 2 pathogenic mutation sites 1 in CREBBP (rs587783505) and 1 in NF1 (rs915463951). NF1 also has a mutation of conflicting interpretations of pathogenicity (rs377662483) (Table 3). These results suggest their potential role in the pathogenesis of OSCC.

**Table 3:** Mutation information of 12 genes mutated in all samples.

Genes	Mutation Information						
	Sample 1 (2FA)	Sample 2 (3FA)	Sample 3 (4FA)	Sample 4 (6FA)	Sample 5 (7FA)	Sample 6 (8FA)	Sample 7 (10FA)
LRP1B	rs781348384 rs758423489 rs752953811 rs776629743 rs758715859 rs778319539 16 not reported	1 not reported	3 not reported	2 not reported	rs759169172 rs977321974 rs150366199 33 not reported	4 not reported	rs756605370 rs762036339 rs12990449 21 not reported
SETD2	rs765767219 rs537154191 rs762731738 12 not reported	1 not reported	rs139016283 rs775039657 2 not reported	2 not reported	rs753817699 rs55704515 rs1019451348 18 not reported	rs779851402 2 not reported	12 not reported
CREBBP	rs587783505 13 not reported	1 not reported	rs765721268 1 not reported	rs776419949 4 not reported	10 not reported	rs756972103 rs55960450 rs181646656 1 not reported	rs200566758 rs1048314482 rs130015 12 not reported
IGF2R	rs748380169 8 not reported	1 not reported	1 not reported	1 not reported	rs1018194928 rs766388482 rs368697729 rs629849 rs773403015 17 not reported	1 not reported	rs771501337 rs761398978 rs629849 3 not reported
PTPRT	rs997633979 rs2867655 12 not reported	rs2867655	rs2867655	rs2867655 1 not reported	rs2867655 10 not reported	rs2867655	rs752743357 rs2867655 rs41310016 5 not reported
ARID2	rs768629455 7 not reported	1 not reported	2 not reported	2 not reported	rs896129362 rs764179676 rs531953821 rs748381246 rs757492254 13 not reported	2 not reported	8 not reported
EPHB6	5 not reported	4 not reported	3 not reported	6 not reported	rs754690459 rs573964960 7 not reported	4 not reported	rs768967254 rs759946110 rs370383805 4 not reported
TET2	rs764470309 5 not reported	rs6843141 1 not reported	rs765414159 1 not reported	rs984803703	rs747747352 rs777027345 13 not reported	rs17253672 2 not reported	rs768670105 7 not reported
NF1	rs750603086 20 not reported	1 not reported	rs915463951 2 not reported	6 not reported	rs876659417 rs377662483 rs915463951 18 not reported	rs757512142 4 not reported	rs267604794 rs1004943800 rs876658849 10 not reported
ESR1	3 not reported	1 not reported	rs367647625	1 not reported	rs751068624 2 not reported	1 not reported	2 not reported
CIC	rs746217053 4 not reported	1 not reported	rs543498277	1 not reported	rs962526448 rs370252719 9 not reported	1 not reported	rs563224468 rs587778206 9 not reported
CBL	2 not reported	1 not reported	1 not reported	1 not reported	rs368326846 10 not reported	1 not reported	2 not reported

### Immunohistochemical analysis

**Figure 2:**
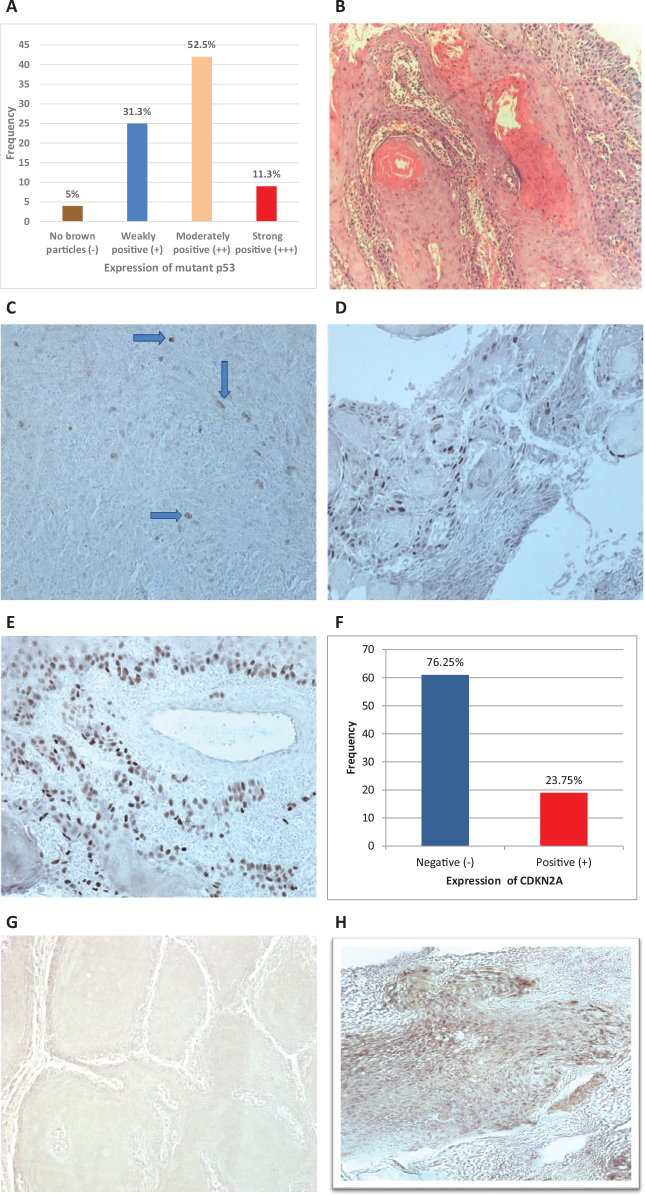
Analysis of immunohistochemical staining of OSCC Naswar users with mutant Tp53 and CDKN2A. (A) Frequency and percentage of mutant TP53 expression in OSCC cases. (B) H & E photomicrograph of OSCC without IHC staining, magnification, 10x. (C) OSCC showing weak mutant p53 (+) expression (IHC, magnification, 20x). (D) OSCC showing moderately positive mutant p53 (++) expression (IHC, magnification, 20x). (E) OSCC showing strong positive mutant p53 (+++) expression (IHC, magnification, 20x). (F) Frequency and percentage of CDKN2A expression in OSCC cases. (G) OSCC showing negative CDKN2A expression (IHC, magnification, 20x). (H)OSCC showing strong positive and diffuse CDKN2A cytoplasmic and nuclear expression (IHC, magnification, 20x).

The immunohistochemical expression of mutant p53 and CDKN2A was investigated in 80 OSCC cases of Naswar users. The clinico-pathological variables of these patients are summarised in Table 4. Mutant p53 was moderately expressed in 52.5% cases and 31.3% of the cases were weakly positive (Figure 2A). The immunohistochemical evaluation of CDKN2A revealed positive expression in 23.7% cases while 76.2% cases showed negative CDKN2A expression (Figure 2F).

**Table 4:** Clinico-pathological variables of 80 patients.

Clinico-pathological variables	Total no of patients (*N* = 80) (%)
**Age in years**	
Mean	56.98
Standard deviation (SD)	± 12.94
Range	30–85
**Gender**	
Male	59 (73.0)
Female	21 (26.0)
**Site**	
Buccal mucosa	28 (35.0)
Alveolar ridge	20 (25.0)
Retro molar trigone	15 (18.3)
Lat Border Tongue	9 (11.3)
Lip	8 (10.0)
**Risk habits**	
Snuff only	75 (93.0)
Snuff with other products	5 (6.3)
**Duration of use in years**	
1–10	9 (11.0)
11–20	26 (32.0)
> 20	45 (56.0)
**Status**	
Present user	63 (78.7)
Ex user	17 (21.2)
**Duration of lesion**	
< 1 month	2 (2.5)
1–6 months	66 (82.5)
> 6 months	12 (15.0)
**Degree of differentiation (WHO)**	
Well	63 (78.7)
Moderate	13 (16.2)
**Variants**	
Adenoid SCC	1 (1.25)
Basaloid SCC	1 (1.25)
PapillarySCC	1 (1.25)
Verrucous SCC	1 (1.25)
**Stage (AJCC 8th edition)**	
I	12 (15.0)
II	10 (12.5)
III	15 (18.7)
IV	43 (53.7)

WHO: World Health Organization.

The correlation of expression of mutant TP53 and CDKN2A with variables (age, gender, site, duration of use, grade, Tumour, Node, Metastasis (TNM) staging) was assessed by applying Pearson chi-square test. The statistics revealed a significant correlation of mutant TP53 and CDKN2A expression with TNM staging of OSCC patients (Table 5).

**Table 5:** Correlation of expression of mutant TP53 and CDKN2A with clinic-pathological parameters of OSCC patients.

Expression		Age	Gender	Site	Duration of use	WHO Grade	TNM staging
**Mutant TP53**	Value	1.671^a^	1.926^a^	9.298^a^	16.667^a^	10.117^a^	47.316^a^
	Asymp. Sig. (2-sided)	0.644	0.588	0.677	**0.054**	0.812	**0.000**
**CDKN2A**	Value	1.359^a^	0.348^a^	2.260^a^	4.040^a^	4.148^a^	7.842^a^
	Asymp. Sig. (2-sided)	0.244	0.555	0.688	0.257	0.528	**0.049**

WHO: World Health Organization.

## Discussion

The exposure to environmental risk is modifying the genetic aetiology of OSCC to a greater extent. Therefore, the researchers are diligently investigating the mutational profile of OSCC for understanding the genetic diversity and the innovation of targeted therapy. Due to scarcity of the research on mutational landscape of OSCC with single contributing risk factors, the present study is performed to identify more somatic variants associated with the occurrence of OSCC to better treat the disease.

Regarding the mutational profile of OSCC associated with and without risk factors, we found a series of studies conducted in different parts of the world. Stransky and his colleagues were first to uncover mutational spectrum of HNSCC in 2011 by WES. The most commonly mutated gene was TP53 (62%) followed by NOTCH1 (14%), CDKN2A (12%), FAT1 (12%), and CASP8 (8%), and the type of variant detected was deletion in three genes (TP53, CDKN2A, NOTCH1). These observations are comparable to our findings with deletion identified in NOTCH1 and FAT1 genes [12]. In Japanese population, with the help of targeted amplicon sequencing, the frequent mutated genes found to be in OSCC were TP53 (61.7%), NOTCH1 (25.5%), and CDKN2A (19.1%). Among detected variants, splice site also was noticed in these genes. We recognised same variant in TP53 and CDKN2A [26]. Moreover, Lin et al. unveiled TP53 (62%), FAT1 (40%) NOTCH1 (28%), and CASP8 (22%) as significantly mutated genes which are slightly different from our evaluation as CDKN2A has not been shown in top 20 altered genes in their samples. The somatic variants detected were missense, frameshift, splice site, and stop gain [27]. Various studies presenting mutational profile with respect to TSGs of OSCC are in line with our findings [10, 28–34], while opposing results were documented by Liu et al. [35].

The WES of 15 OSCC patients consuming Arabian snuff locally known as shammah (a form of SLT) has revealed novel somatic variants in driver genes such as CSMD3, OSMR, NOTCH3, FADD, CRB1, TRMP2, and CLTCL1; amplification of oncogenes and deletion of SMARCC1, a TSG in addition to previously confirmed genes in OSCC [13]. The exposure of the patients chewing areca nut famous in South and South East Asia exhibit distinctive mutational profile stating new mutations in ATG2A, WEE1, DST, TSC2 along with already known genes [16]. Similarly, Alshahrani et al. performed genomic analysis of khat users which is basically a plant and its leaves are chewed as a cultural tradition especially in Saudi Arabia. It contains alkaloid cathinone that might be implicated in carcinogenicity. They described somatic mutations in five cancer related genes (ARID1A, ARID2, PIK3CA, MLH1, and TP53) [14]. From the study of patients chewing betel quid, the genomic landscape demonstrated different group of mutated genes with higher rate of mutations found in TP53, RASA1, BRCA2, NOTCH1, CDKN2A, PCMTD1, and PGM5 [15]. Pansare et al. identified genetic alteration associated with SLT in PCLO, FAT3, SYNE2, and PIK3CA in OSCC cell lines [36].

Our research has indicated a distinguishing panel of TSGs which was seen to be mutated in all samples (100%) consuming Naswar. This panel is comprised of LRP1B, SETD2, CREBBP, IGF2R, PTPRT, ARID2, EPHB6, TET2, NF1, ESR1, CIC, and CBL. The COSMIC database has also ranked LRP1B in top 20 frequently mutated genes. Several reports have shown the occurrence of LRP1B mutation in cell lines of tongue cancer and head and neck squamous cell carcinoma (HNSCC) [29, 37]. Recently, LRP1B single nucleotide polymorphism (rs10496915, rs431809, and rs6742944) were assessed in OSCC patients with diabetes mellitus [38]. The deletion of LRP1B was also found to be enriched in recurrent cases of HNSCC after treatment which might be considered as probable biomarker for treatment of resistant cases [39]. However, SETD2 (histone lysine methyltransferase SETdomain containing 2) mutations has been implicated in different cancers [40], but studies showing its occurrence in oral cancer are scarce [41]. Further, Wang et al. have confirmed mutation of SEDT2 in HNSCC [42]. The project of Indian genomic team on 50 cases of gingivo-buccal OSCC revealed ARID2 as significantly mutated gene [43]. Correspondingly, Ghias et al. have identified SNVs in the same gene in HNSCC [11]. Among 30 cases of OSCC, rate of mutation in ARID2 gene reported by Das and his co-workers is about 6% [44]. The evidence from genomic analysis of tongue carcinoma has uncovered CREBBP mutation which is in line with our results [45]. Our findings are supported by two independent studies observing CREBBP mutation in HNSCC [42, 46]. Recently, CIC (capicua transcriptional repressor) has been listed in a set of unique genes, the alteration of which may contribute to the recurrence of OSCC [47]. The genetic changes in CBL that belongs to a family of RF (ring finger) ubiquitin ligases in solid tumours is known [48], while investigation of the same gene in OSCC samples has led to the conclusion that CBL genetic alteration are infrequent in this type of neoplasia [49]. In Taiwan population, WES using multiple pipelines of mutant calling has revealed somatic mutation of IGF2R in oral cancer [27]. Regarding genetic changes in PTPRT, we determined a number of somatic variants equivalent to other studies [27, 29, 50]. Comparing mutational outline of recurrent and non-recurrent cases of oral cavity cancer, aberration of NF1 gene was seen in maximum patients [47]. A study has shown the presence of NF1 gene in OSCC cell lines [29]. There have been reports on TET2 epigenetic changes, and currently a correlation of TET2 DNA methylation with OSCC has been established [51]. But due to limited research on this aspect, we were able to find TET2 modification at genetic level in cell lines only [29]. Further, the information with respect to somatic mutations in EPHB6 and ESR1 in OSCC is also inadequate; while pooling of these mutations is well documented in other malignant tumours [52, 53]. Regarding EPHB6 gene, Kolegova et al. reported mutations in recurrent tongue cancer; while our samples comprised of primary tumour, identified with these mutations [54].

The present analysis has demonstrated higher prevalence of C/T and G/A base change among single nucleotide variants. Several studies highlighted the analogous conclusion with elevated rate of C/T substitution [29, 31, 55–57]. With reference to the site, OSCC of tongue also harboured C/T transitions in more samples as compared to others [45]. Interestingly, C/T nucleotide change was observed in Arabian inhabitants addicted to the use of SLT [13]. In context of risk factors, dominant pattern of C/T and G/A transitions have been reported in betel quid users [15]. On the basis of these findings, we can validate the frequent occurrence of C/T base change in OSCC patients exposed to SLT further verifying our results. Patel et al. in contrast revealed C:G > A:T as highly contributing base change in cohort of chewers [32], while Stransky et al. and Indian team of genomics showed maximum portion of G > T and C > G transversions respectively, in patients with history of tobacco [12, 43].

Most of the cases (52.5%) showed moderate positive expression of mutant TP53. The parameters in which statistically significant difference was observed were clinical stage and duration of use of Naswar. Li et al. aimed to evaluate the same correlation by examining a positive expression of mutant TP53 with clinical stage. They also concluded a positive expression regarding degree of differentiation of OSCC (well, moderate, and poor) conflicting to what we have observed in our cases [24]. Among OSCC cases, another study assessed an increase in TP53 positive cells with the increasing grades from well to poorly differentiated OSCC [58]. The relationship of TP53 protein with unfavourable histopathological characteristics was also recognised in other study [59]. Comparing mutations in TP53 and its protein expression, Hyodo et al. found its abnormal accumulation in nucleus on immunohistochemistry where mutations were detected in cases by next generation sequencing (NGS) [60]. We found that the majority of the cases (76.25%) were negative for the CDKN2A expression on immunohistochemistry in OSCC samples. With respect to correlation, a significant difference was established between CDKN2A expression and clinical stage. The immunohistochemical staining of CDKN2A protein was present in only 10% cases of OSCC in one of the studies displaying analogous results; however, the association between variables and protein expression was not significant [25]. Shyamsundar et al. performed both targeted exome sequencing of this gene for somatic mutations and its immunohistochemical evaluation. They found mutations in 4 samples and recorded p16 overexpression in only 21.4% OSCC patients observing significant correlation with grades [61]. Another study concluded its low expression in OSCC by comparing means of P16 positive cells in two groups (dysplasia and carcinoma) [62]. In addition, CDKN2A overexpression was not identified in 95% of oral tongue cancer along with insignificant correlation of its expression with clinical features [63]. Anwar et al. spotted CDKN2A nuclear and cytoplasmic staining in total 19 out of 50 cases also signifying its low expression in our population [64].

Inevitably, the present study has several limitations. Firstly, the sample size is relatively small which might explain the low occurrence of some of the frequently mutated genes in OSCC. However within limited resources, it was important to provide basic background information ensuing large cohorts. Secondly, we performed WES which focuses on coding regions of DNA and can detect SNV and indels. Therefore, structural variants and variants in non-coding regions of DNA remain undetected that might have key roles in the development of OSCC and targeted therapy.

## Conclusions

In summary, our study is the first of its kind reporting mutational landscape of OSCC patients with a habit of Naswar use. We investigated a discrete panel of genes (LRP1B, SETD2, CREBBP, IGF2R, PTPRT, ARID2, EPHB6, TET2, NF1, ESR1, CIC, and CBL) found to be mutated in all OSCC patients. Compared to dbSNP, all of these 12 TSGs showed mutations not previously reported. Regarding mutational information, we recognised PTPRT mutation (rs2867655) in all samples. IGF2R (rs629849) was present in 2 samples only. However, further suitable studies are required to confirm the role of PTPRT and IGF2R in the pathogenesis of OSCC.

## Data Availability

The datasets used and/or analysed during the current study are available from the corresponding author on reasonable request.
